# Incidental tumor necrosis caused by the interventional alteration of hepatic arterial flow in patients with advanced hepatocellular carcinoma

**DOI:** 10.1007/s12328-014-0542-y

**Published:** 2014-12-07

**Authors:** Eiichiro Suzuki, Yoshihiko Ooka, Tetsuhiro Chiba, Kazufumi Kobayashi, Naoya Kanogawa, Tenyu Motoyama, Tomoko Saito, Sadahisa Ogasawara, Akinobu Tawada, Osamu Yokosuka

**Affiliations:** Department of Gastroenterology and Nephrology, Graduate School of Medicine, Chiba University, 1-8-1 Inohana, Chuo-ku, Chiba, 260-8670 Japan

**Keywords:** Hepatocellular carcinoma, Hepatic arterial infusion chemotherapy, Alteration of blood flow, Replaced right hepatic artery

## Abstract

Hepatic arterial infusion chemotherapy (HAIC) is one of the approaches used to treat advanced hepatocellular carcinoma (HCC). Here, we describe 2 cases involving unexpected tumor necrosis after interventional alteration of the hepatic arterial flow during implantation of a port-catheter system for HAIC. Case 1 involved a 42-year-old man with diffuse HCC accompanied by a tumor thrombus in the main trunk of the portal vein. After the right hepatic artery (RHA) derived from the superior mesenteric artery (SMA) was occluded by coils, a port-catheter system was successfully implanted using the gastroduodenal artery (GDA) coil method. The next day, he developed a fever and had right upper abdominal pain. A marked increase in transaminase and lactate dehydrogenase levels was observed. Contrast-enhanced computed tomography (CT) showed tumor necrosis in both the parenchymal tumor and portal vein tumor thrombus. Case 2 involved a 62-year-old man diagnosed with a large HCC located in segments VII and VIII of the liver and abdominal lymph node metastasis. As in case 1, angiography revealed the RHA branched from the SMA. After the replaced RHA and right gastric artery were embolized with coils, a port-catheter system was successfully implanted. Although he showed neither clinical symptoms nor abnormal laboratory data the next day, contrast-enhanced CT revealed tumor necrosis in a large part of the HCC lesion. In conclusion, careful attention is required in the interventional alteration of hepatic arterial flow for implantation of a port-catheter system for HAIC against advanced HCC.

## Introduction

Hepatocellular carcinoma (HCC) is the sixth most common cancer worldwide and the third leading cause of death from cancer [1]. HCC usually develops in chronically damaged liver caused by hepatitis viral infection, alcoholic abuse, or nonalcoholic fatty liver disease [2]. Recent innovations in diagnostic tools and therapeutic procedures for HCC have enabled early diagnosis and curative treatments such as hepatic resection and liver transplantation. In addition, non-surgical treatments such as radiofrequency ablation and transcatheter arterial chemoembolization have been applied for unresectable HCC without macrovascular invasion (MVI) and distant metastasis [3]. MVI such as portal vein tumor thrombus (PVTT) is a characteristic of advanced HCC [4]. The prognosis of patients with HCC complicated with PVTT is extremely poor, and the median survival time of these patients is reported to be only several months [5, 6]. In Japan, sorafenib administration and hepatic arterial infusion chemotherapy (HAIC) are the first-line therapeutic approaches for advanced HCC accompanied by PVTT [7]. To perform HAIC safely and effectively, both unification of hepatic arterial flow and occlusion of gastrointestinal arteries are essential for the implantation of port-catheter system [8].

Herein, we report 2 cases of tumor necrosis caused by the alteration of hepatic arterial flow in the implantation of port-catheter systems for HAIC.

## Case reports

### Case 1

**Fig. 1:**
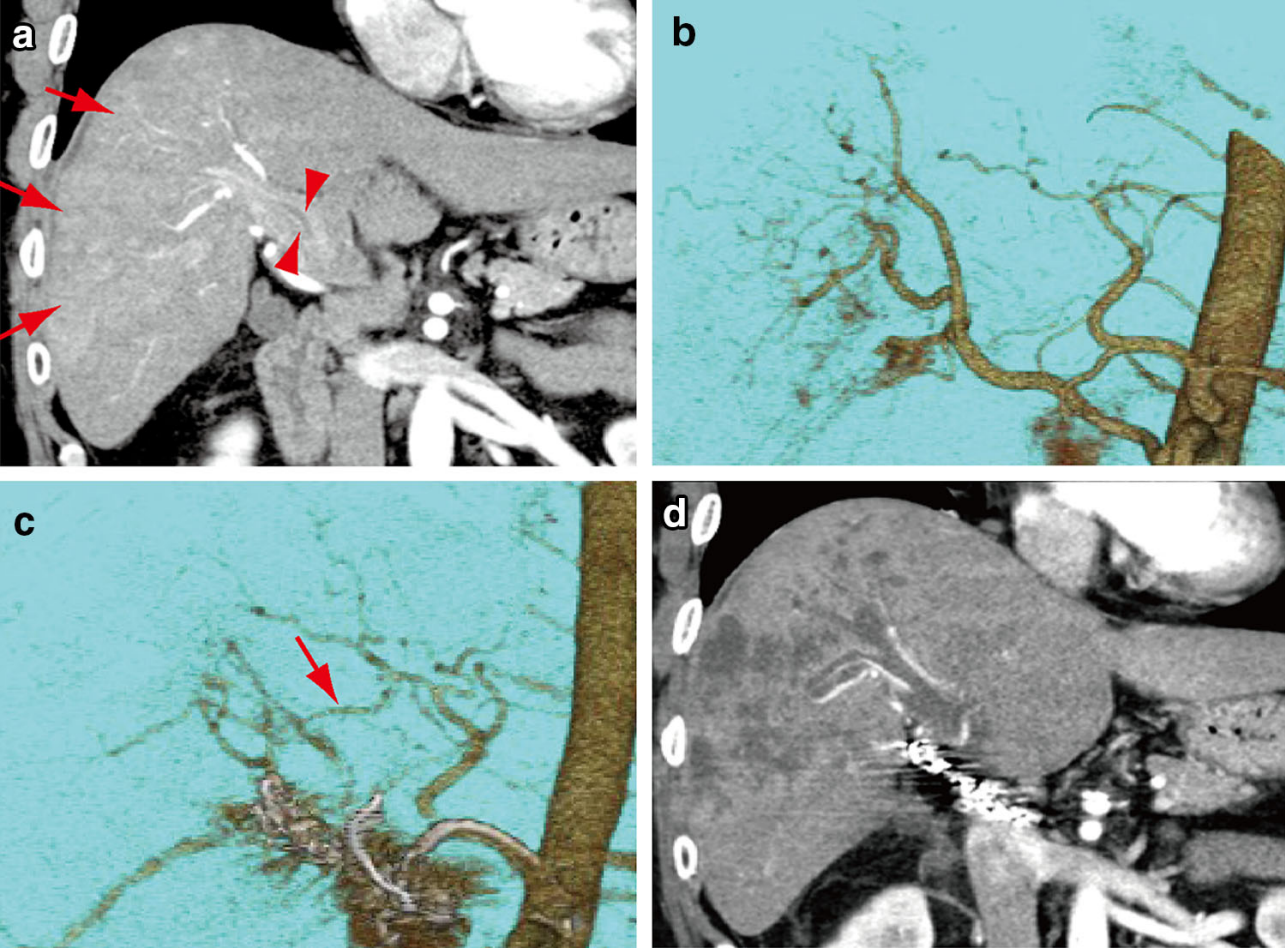
CT findings for case 1. **a** A coronal image in the arterial-dominant phase demonstrates diffuse HCC (*arrows*) with PVTT (*arrowheads*). **b** 3D-reconstructed CT angiography shows RHA branches from the SMA. **c** 3D-reconstructed CT angiography after port-catheter system implantation shows a communicating vessel (*arrow*) between the LHA and the RHA. **d** A coronal image in arterial-dominant phase demonstrates a marked decrease in vascularity of both parenchymal tumors and the PVTT one day after the procedure

A 42-year-old man was referred to our hospital to receive treatment for liver tumors. The patient had not received surveillance for HCC in spite of chronic hepatitis B virus (HBV) infection. On admission, he was symptom-free. A physical examination revealed no signs of hepatosplenomegaly or peritoneal irritation. Although serum levels of alkaline phosphatase and total bilirubin were normal, aspartate aminotransferase (AST, 74 IU/L) and alanine aminotransferase (ALT, 54 IU/L) levels were mildly elevated (Table 1). The patient tested negative for anti-hepatitis C virus (HCV) antibody but positive for hepatitis B surface antigen. The complete blood cell count was normal. Levels of α-fetoprotein (AFP) and des-gamma-carboxy prothrombin (DCP) were markedly increased to 107.3 ng/mL and 97,200 mAU/mL, respectively. Arterial-dominant phase images in contrast-enhanced computed tomography (CT) revealed diffuse hyperdense tumors located in the right lobe of liver (Fig. 1a). These tumors extended into the main trunk of the portal vein. Taking into consideration chronic HBV infection and markedly elevated levels of AFP and DCP, we made a diagnosis of advanced HCC with PVTT.

**Table 1:** Laboratory data for case 1

	On admission	One day after the port implantation
Hematology		
WBC (/μL)	5,800	8,900
RBC (×10^4^/μL)	4.41	3.96
Hb (g/dL)	14.0	12.5
Ht (%)	41.2	37.3
Plt (×10^4^/μL)	20.2	18.4
PT (%)	110	93
Blood chemistry		
AST (IU/L)	74	867
ALT (IU/L)	54	291
LDH (IU/L)	366	6,173
ALP (IU/L)	310	617
T-Bil (mg/dL)	1.3	0.9
BUN (mg/dL)	9	11
Cre (mg/dL)	0.55	0.59
UA (mg/dL)	3.3	3.4
TP (g/dL)	6.6	6.3
Alb (g/dL)	3.1	2.9
NH_3_ (μg/dL)	68	72
Serology		
CRP (mg/dL)	3.9	6.6
HBsAg	+	
HCV-Ab	−	
AFP (ng/mL)	107.3	
DCP (mAU/mL)	97,200

The patient was willing to participate in a clinical trial of sorafenib administration and HAIC. Sorafenib was administrated for 1 week. A port-catheter system was subsequently implanted in the patient using the ‘gastroduodenal artery (GDA) coil method’. Digital subtraction angiography (DSA) revealed that the right hepatic artery (RHA) was derived from the supra mesenteric artery (SMA) (Fig. 1b). After locating the tumors and their feeding arteries, a 5-French catheter with a side vent was inserted into the GDA. The GDA and other arteries supplying the gastroduodenal region were embolized using coils to prevent gastroduodenal ulceration. In addition, the replaced RHA was also occluded using coils to unify the arterial flow. 3D-reconstructed CT angiography showed collateral vessels between the left hepatic artery (LHA) and the RHA (Fig. 1c). The patient experienced abdominal pain and fever the day after the procedure. The laboratory data showed markedly increased levels of AST, ALT, and lactate dehydrogenase (LDH) (Table 1). Because contrast-enhanced CT demonstrated a decline in tumor vascularity in both parenchymal tumors and PVTT, we considered the possibility that interventional alteration of hepatic arterial flow had resulted in tumor necrosis (Fig. 1d). Fluid replacement therapy and administration of antibiotics improved the laboratory data. The data returned to levels similar to those before implantation of the port-catheter system 2 weeks earlier. The levels of AFP and DCP decreased to 21.9 ng/mL and 3,380 mAU/mL, respectively. Subsequently, HAIC with 5-fluorouracil (5-FU) and cisplatin was initiated. The patient is still living 4 months after implantation of the port-catheter system and is now receiving HAIC treatment with sorafenib.

### Case 2

**Fig. 2:**
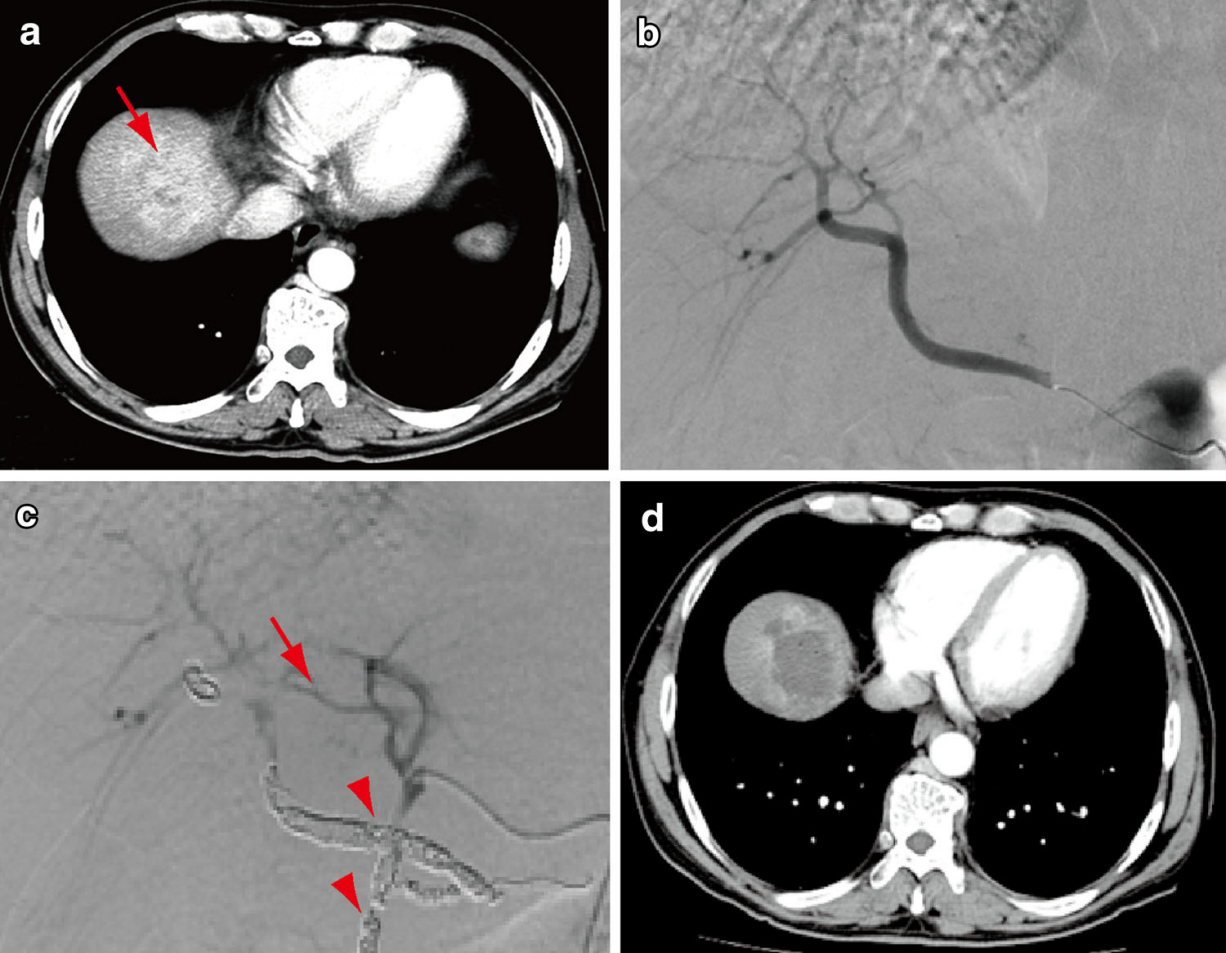
CT and angiography findings for case 2. **a** A CT image in the arterial-dominant phase demonstrates HCC (*arrow*) of approximately 50 mm in diameter in segments VII and VIII. **b** Supra mesenteric arteriography reveals the RHA branches from the SMA. **c** Celiac arteriography after occlusion of the GDA and replaced RHA using coils (*arrowheads*) shows communicating vessel (*arrow*) between the LHA and RHA. **d** A CT image in the arterial-dominant phase after port-catheter system implantation reveals a marked decrease in vascularity of the tumor

A 62-year-old man with liver tumors and abdominal lymph node enlargement was admitted to our hospital for treatment. He had been followed for liver cirrhosis due to alcohol abuse but had not yet developed HCC. He had refrained from drinking for the 5 years before admission. On admission, he was asymptomatic, and physical examination revealed no special findings. Serum levels of AST (48 IU/L) and total bilirubin (T-Bil, 1.3 mg/dL) were mildly elevated. He showed marked thrombocytopenia (7.4 × 10^4^/μL) accompanied by splenomegaly. Serological tests for hepatitis-B and -C were negative. The level of AFP increased to 2,372.0 ng/mL, while the DCP level was almost normal. Arterial-dominant phase images in contrast-enhanced CT revealed hyperdense tumors 50 mm in diameter in segments VII and VIII of the right hepatic lobe (Fig. 2a). These tumors were accompanied by para-aortic lymphadenopathy. Pathological examination of tumor biopsy samples revealed that the tumor was composed of moderately differentiated HCC. Eventually, the patient was diagnosed with HCC with abdominal lymph node metastasis.

Similar to case 1, a port-catheter system was implanted in the patient using the GDA coil method for HAIC. DSA revealed that the RHA branched from the SMA (Fig. 2b). After locating the tumors and their feeding arteries, the replaced RHA was occluded using coils to unify the arterial flow. An intra-arterial catheter with a side vent was inserted into the GDA. Both the GDA and right gastric artery (RGA) were embolized using coils to prevent gastroduodenal mucosal damage. Celiac arteriography successfully revealed blood flow from the LHA to the RHA through the collateral vessels (Fig. 2c). This patient exhibited neither fever nor abdominal pain after the procedure, unlike case 1. Although laboratory data revealed a slightly increased C-reactive protein (CRP), hepatobiliary enzyme levels were normal. Contrast-enhanced CT demonstrated a remarkable decrease in tumor vascularity, indicating tumor necrosis (Fig. 2d). The patient was discharged 7 days after his admission. Because the patient was willing to participate in a clinical trial of sorafenib administration and HAIC, he subsequently received HAIC with 5-FU and cisplatin and sorafenib treatment. He is still living 2 months after implantation of the port-catheter system and is being treated with combination therapy.

## Discussion

HAIC was developed as a therapeutic approach against advanced HCC more than 10 years ago, and the utility and effectiveness of HAIC are widely accepted in Japan [9]. Nonetheless, HAIC is not considered a treatment option for patients with advanced HCC in the guidelines proposed by the American Association for the Study of Liver Diseases (AASLD) and the European Association for the Study of the Liver (EASL) [10–12]. The implanted port-catheter system for HAIC allows the delivery of anticancer drugs to HCC at relatively high concentrations. The toxicity caused by systemic distribution of anticancer drugs can be reduced by the first-pass effect [13]. Arteries supplying the gastroduodenal region such as the GDA and RGA are typically occluded by coils to prevent gastroduodenal ulceration. However, anatomic variations in hepatic arteries are observed frequently [14, 15]. A replaced LHA, branching from the left gastric artery (LGA), is present in approximately 10 % of the population. Similarly, a replaced RHA, derived from the SMA, is also observed in approximately 10 % of the population. In these cases, it is necessary to redistribute the hepatic arterial flow from multiple arteries into a single artery to perform HAIC safely and effectively.

Both patients presented here had a replaced RHA (branching from the SMA) and exhibited marked tumor necrosis after the alteration of hepatic arterial flow for implantation of the port-catheter system. Both patients received first-time treatment of HCC. In addition, their tumors were located in the upper section of the right hepatic lobe. We performed implantation of the port-catheter system for HAIC in 33 patients with HCC between January 2008 and May 2014. Although 4 of the 33 patients had a replaced RHA, incidental tumor necrosis was only observed in these 2 patients. Although many of the previous cases had received transcatheter arterial chemoembolization (TACE) before the HAIC, it is not uncommon for recent cases to undergo HAIC as the initial treatment for HCC. In these TACE-naïve cases with a replaced RHA, blood supply to tumors might be heavily dependent on the RHA.

Novel modalities such as perfusion CT are utilized to accurately determine the changes in blood flow [16]. Although we could not perform perfusion CT in these cases, we calculated CT values as an alternative. The CT values of non-tumorous tissues in the right hepatic lobe, after the implantation of the port-catheter system, decreased by approximately 10 HU compared with those before the procedure. However, those in the left hepatic lobe showed no remarkable changes. Intrahepatic collaterals between branches of the hepatic arteries have been reported to develop immediately after arterial occlusion [17]. In line with this observation, angiography after the alteration of hepatic arterial flow revealed a blood supply from the LHA to the RHA through connecting vessels. It is possible, however, that the blood supply to the right lobe after the alteration of arterial flow may have been insufficient compared with that supplied by the replaced RHA. A decrease in the hepatic arterial blood supply caused by subintimal injury results in necrosis of HCC tissues [18]; therefore, occlusion of the replaced RHA by coils may cause a rapid and drastic decrease in the blood supply to tumors, resulting in unexpected tumor necrosis in our cases.

It has been reported that arterial flow is frequently supplied by the right inferior phrenic artery (RIPA) after occlusion of the hepatic artery [19, 20]. Although no remarkable changes in the diameter of the RIPA were observed in CT images before and after the implantation of the port-catheter system, CT angiography (CTA) images obtained via the catheter placed at the RIPA before and after the alteration of hepatic arterial flow might be of importance to evaluate the changes in the flow of RIPA in such cases. It is possible that the inadequate blood supply from the neighboring extrahepatic arteries, including the RIPA, after occlusion of the RHA is partly responsible for the incidental tumor necrosis. In addition, some coils appeared to be placed near the bifurcation of the anterior branch and posterior branch in case 1. Celiac arteriography, after port-catheter system implantation, visualized both the anterior and posterior branch, but this procedure might have also affected the blood supply to the right hepatic lobe through the vessels connecting the anterior branch and posterior branch. Thus, it is necessary to pay attention to the extent of coil embolization.

Neovascularization is a major characteristic of HCC. Tumor vessels usually have an irregular diameter and an abnormal branching pattern [21, 22]. In addition, tumor vasculature lacks a complete basal membrane and pericyte cover. Thus, the collaterals that develop between the LHA and tumor vessels are likely to be insufficient compared with those between the LHA and non-tumorous tissues in the right hepatic lobe. Sorafenib is an oral multi-kinase inhibitor that mainly blocks RAS/RAF/MEK/ERK kinase signals and receptor tyrosine kinases associated with angiogenesis, including VEGFR-2/-3 and PDGFR-β. This agent thereby inhibits tumor cell proliferation and interferes with tumor angiogenesis [23]. Case 1 had been treated with sorafenib for 1 week. Although no remarkable changes in tumor enhancement were observed between transvenous contrast-enhanced CT images before sorafenib administration and CTA images at the time of the port-catheter system implantation, it is possible that intratumoral blood flow had already decreased compared with that before the sorafenib administration [24, 25]. It is also possible that the blood supply to HCC tissues through the collaterals may have been inhibited after the RHA occlusion.

Tumor lysis syndrome (TLS) is caused by the rapid destruction of cells after an effective cancer treatment. In TLS, degradation products released from the nuclei and cytoplasm of the tumor cells often cause life-threatening complications, including disseminated intravascular coagulation (DIC) and acute renal failure. TLS is reported not only in patients who have hematological malignancies but also in those with solid organ tumors including HCC [26, 27]. Case 1, but not case 2, showed a marked increase in the levels of transaminases and LDH. Because the patient did not exhibit severe TLS complications such as DIC, and because the elevated enzyme levels returned to baseline after conservative management, this might be attributable to hepatic injury rather than TLS. A previous report showed that tumors with a large diameter, high sensitivity to the treatment, impaired renal function, and dehydration and hypercalcemia before treatment are associated with the onset of TLS [28]. Therefore, HCC patients with these risk factors should be closely monitored after port-catheter implantation involving alteration of hepatic arterial flow.

In conclusion, we have observed 2 cases demonstrating remarkable tumor necrosis after modification of hepatic arterial flow. To ascertain the link between the modification of hepatic arterial flow and tumor necrosis, further research is necessary, with a larger number of patients. Because HCC patients treated with HAIC frequently exhibit a decreased hepatic reserve, alteration of hepatic arterial flow requires special attention during implantation of a port-catheter system.
